# Microleakage Assessment of Modified Resin Infiltration With Zinc Oxide (ZnO) and Magnesium Oxide (MgO) Nanoparticles on Artificial Enamel Caries Lesion: An In Vitro Study

**DOI:** 10.7759/cureus.66617

**Published:** 2024-08-11

**Authors:** Dler A Khursheed, Aras M Rauf, Hadi M Ismail

**Affiliations:** 1 Periodontics, College of Dentistry, University of Sulaimani, Sulaymaniyah, IRQ; 2 Pedodontics and Community Oral Health, College of Dentistry, University of Sulaimani, Sulaymaniyah, IRQ; 3 Orthodontics, College of Dentistry, University of Sulaimani, Sulaymaniyah, IRQ

**Keywords:** nanoparticles, mgo, zno, methylene blue, microleakage, icon resin infiltrant

## Abstract

Background

Incorporating nanoparticles into resin infiltrant (RI) can alter its physical properties, including microleakage. This study aimed to examine the microleakage properties of RI modified with 2.5% and 5% zinc oxide (ZnO) and magnesium oxide (MgO) nanoparticles on artificially induced carious lesions (ACLs) in bovine teeth.

Materials and methods

Modified RIs were applied to five sound and 30 ACLs on bovine incisors. They were divided into seven groups based on the procedure (Group 1: sound enamel (SE), Group 2: artificial caries lesion only (ACL), Group 3: RI, Group 4: RI+ 2.5% ZnO, Group 5: RI + 5% ZnO, Group 6: RI + 2.5% MgO, Group 7: RI + 5% MgO). The samples were subjected to 5000 thermal cycles and then exposed to methylene blue solution for 24 hours in an incubator. The teeth were sectioned and examined with a stereomicroscope to assess the depth of methylene blue penetration (MBP), quantifying microleakage according to the following scoring: 0 = no penetration, 1 = outer half of enamel, 2 = inner half of enamel, 3 = outer half of dentin, and 4 = inner half of dentin. An ANOVA test was conducted to assess the significance of differences among the groups. Post-hoc tests, including Tukey's HSD (honestly significant difference) test, were performed to identify specific group differences and determine which differences were statistically significant.

Results

SE samples (Group 1) showed minimal penetration, with 100% scoring 1, indicating high resistance to MBP. The ACL group had significant penetration, with 77.78% scoring 3 and 22.22% scoring 2. The RI group completely prevented MBP, with all samples scoring 0. Both RI+2.5% ZnO (Group 4) and RI+2.5% MgO (Group 6) were highly effective, with 88.89% scoring 0 and 11.11% scoring 1. RI+5% ZnO (Group 5) and RI+5% MgO (Group 7) also prevented MBP but had slightly higher minimal penetration, with 77.78% scoring 0 and 22.22% scoring 1. Significant differences were observed between the ACL group and all other groups, underscoring the effectiveness of RI treatments. No significant differences were found between RI+ZnO and RI+MgO at both concentrations, indicating similar effectiveness.

Conclusions

The study demonstrated that RI modified with 2.5% and 5% ZnO and MgO nanoparticles effectively reduced microleakage in ACLs on bovine teeth compared to untreated lesions. These modifications significantly inhibited MBP, particularly in enamel and dentin, indicating their potential to enhance the durability and effectiveness in clinical applications. This research emphasizes the promising role of nanoparticle-modified RI in minimizing microleakage and optimizing treatment outcomes. Further research is needed to confirm these findings and refine protocols.

## Introduction

Dental caries stands as the most common chronic disease globally [1]. Approximately, 2.4 billion individuals globally suffer from persistent dental caries [2]. This non-transmissible condition arises from complex interactions involving a biofilm, dietary influences, and multiple other factors. The disease's progression is characterized by alternating phases of demineralization and remineralization of the dental hard tissues. A cavity will form when the extent of demineralization exceeds that of remineralization. This balance between demineralization and remineralization is critical in determining whether dental caries will develop [3]. Additionally, a variety of biological, behavioral, psychosocial, and environmental elements contribute to its onset and development [4-6].

Initial proximal caries pose significant diagnostic and therapeutic dilemmas [7]. Advances in dental technology in the detection of initial caries lesions have emerged recently as visual and radiographic methods for detecting such early enamel mineral loss are not very consistent [8-10]. Methods like fluorescence and transillumination, as well as advanced tools like optical coherence tomography (OCT), laser fluorescence, and quantitative light-induced fluorescence (QLF), are effective for early caries detection. Optical methods like fluorescence and transillumination are particularly successful in identifying initial caries stages [10]. On the other side, advancements, in adhesive bioactive and bio-interactive restorative materials have enabled the conservation of maximum tooth structure with minimal surgical intervention [11,12].

Icon resin infiltrate (RI) (DMG Dental-Material Gesellschaft mbH, Hamburg, Germany) is a light-curable, low-viscosity solution without fillers, formulated with triethylene glycol dimethacrylate (TEGDMA) dissolved in ethanol and camphoroquinone as the photoinitiator [13]. RI operates by infiltrating deep carious lesions via capillary action, effectively sealing enamel pores to halt caries progression [14-16]. It is indicated for lesions not extending beyond the middle third of the dentin [17]. RI lacks antibacterial properties, which limits its ability to inhibit bacterial growth [18]. Additionally, resin-based dental fillings are prone to bacterial colonization and polymerization shrinkage at the margins, potentially causing gaps at the tooth-resin interface, and increasing the risk of secondary caries and periodontal issues [19-21]. A recent study has shown promising results in sealing artificial white lesions and pits and fissures too [22,23].

Zinc oxide (ZnO) and magnesium oxide (MgO) nanoparticles demonstrate effective antibacterial activity against *Streptococcus mutans*, while also maintaining biocompatibility and possessing advantageous optical properties [24-27]. The efficacy of preventive and restorative dentistry hinges on securing a complete seal in dental restorations. Incorporating antibacterial agents into RI, without compromising sealing property, could offer significant benefits. Yet, a major challenge remains microleakage, characterized as the clinically undetectable transfer of bacteria, fluids, molecules, or ions between the tooth and the applied restoration material [28]. Therefore, the aim of this study was to examine the microleakage property of modified RI with 2.5% and 5% of ZnO and MgO nanoparticles on the artificially induced caries lesions (ACLs) in bovine teeth.

## Materials and methods

This study was conducted on bovine incisors in the College of Dentistry, University of Sulaimani, Sulaymaniyah, Iraq. The study was approved by the Scientific and Ethics Committee of the College of Dentistry, University of Sulaimani (Issue No. 46/21 on 01/09/2021).

Sample

**Figure 1:**
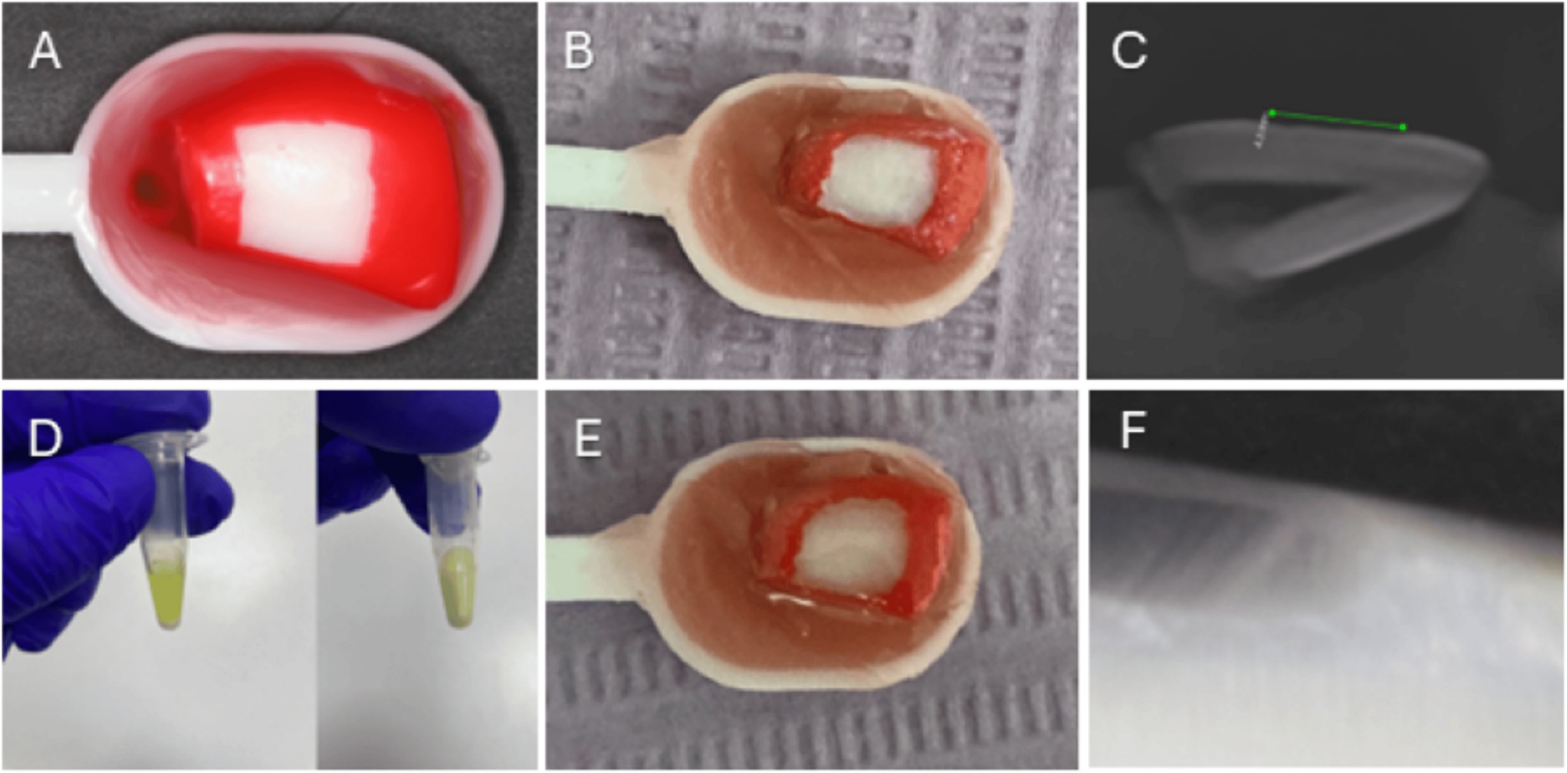
(A) Preparation of the bovine tooth for ACL embedded in wax after 4x4 mm exposing window with nail varnish; (B) Clinical view of ACL; (C) CBCT image showing ACL; (D) Mixture of modified RI with NPs; (E) Infiltration of ACL with modified RI; (F) Microleakage assessment of RI after 5000 thermocycles. ACL: artificially induced caries lesion; RI: resin infiltrant; NP: nanoparticle; CBCT: cone-beam computed tomography

A total of 38 bovine incisors were obtained from a slaughterhouse. The teeth were carefully examined under a stereomicroscope at a magnification of 100x to ensure that they were free from any stains, decay, or cracks. To clean the teeth, ultrasonic treatment was used to remove debris, soft tissue remnants, and calculus. After cleaning, the teeth were stored in deionized water. Then the samples were divided into seven groups, each consisting of five teeth: Group 1: sound enamel (SE), Group 2: ACL, Group 3: RI, Group 4: RI+ 2.5% ZnO, Group 5: RI + 5% ZnO, Group 6: RI + 2.5% MgO, and Group 7: RI + 5% MgO. Additionally, three extra samples were utilized prior to the main sampling to evaluate the ACLs using cone-beam computed tomography (CBCT) with the CS 9600 CBCT scanner (Carestream Dental LLC, Atlanta, Georgia, United States) (Figure 1C).

Specimen preparation

The crown of each tooth was removed using a low-speed handpiece with flat discs and copious water irrigation. After that, the teeth were temporarily stored in 75% ethanol for 24 hours to eliminate any bacteria and viruses. The samples were cleaned with a bristle brush and rinsed with water spray. Enamel windows measuring 4 × 4 mm were created around the middle one-third on each labial surface of the crowns by applying double layers of acid-resistant nail varnish to the surrounding surfaces. Then the samples were embedded in wax in the stool test spoon (Figure 1A). This setup facilitated the manipulation of the samples during the experiment. The tooth samples were attached to the stool test spoon with wax to securely hold them in the demineralization and remineralization solutions within the containers [29]. All samples were stored in distilled water at a temperature of 37°C.

ACL formation

Each sample was immersed in 30 ml of a demineralizing solution (Biochem Chemopharma Company, Cosne-Cours-sur-Loire, France), containing 1.5 mM calcium chloride (CaCl_2_), 0.9 mM sodium dihydrogen phosphate (NaH_2_PO_4_), and 0.15 M potassium chloride (KCl), with pH 7.0 adjusted with 1 M potassium hydroxide (KOH), for six hours per day over a period of seven days. Following this demineralization phase, the samples were immersed in a remineralizing solution (Biochem Chemopharma Company), consisting of 1.5 mM CaCl_2_, 0.9 mM NaH_2_PO_4_, 0.15 M KCl, and 0.25 ppm fluorine (F) with a pH 7.0 adjusted with 1M KOH. All specimens were subsequently stored in distilled water at 37°C until further experimentation (Figure 1B) [30].

Characterization of the nanoparticles by X-ray diffraction (XRD)

The crystalline structure of the nanoparticles was examined using XRD with a PANalytical X’Pert PRO (Cu Kα = 1.5406 Å) (Max-Planck-Gesellschaft, Munich, Germany) at a scanning rate of 1°/minute within the 2θ range of 20-80°.

Application of modified RI

Modified RI of 2.5% and 5% of ZnO and MgO nanoparticles were prepared by mixing the nanoparticles with the Icon RI in an Eppendorf tube and then vibrated for 180 seconds in an amalgamator (Table 1). The mixing ratios were by prepared weight. The Icon RI and the modified Icon RI were then applied to the prepared teeth according to the manufacturer’s instructions. The prepared areas (4 X 4 mm windows) were etched with Icon Etch (DMG Dental-Material Gesellschaft mbH) for two minutes; then water sprayed for 30 seconds and dried. Icon Dry (DMG Dental-Material Gesellschaft mbH) was applied for 30 seconds and dried, and finally, the Icon RI was applied for three and one minutes and then subsequently light-cured for 40 seconds using high-intensity 2200-2400 W/cm^2^ curing light (Baolai Medical Instrument Co. Ltd, Nanning, Guangxi, China) each time after excess material was removed (Figures 1D, 1E). All the samples were properly polished using 1500- and 2000-grit silicon carbide (SiC) paper and finished.

**Table 1:** Compositions and manufacturers of materials used ZnO: zinc oxide; MgO: magnesium oxide

Materials and equipment	Composition	Manufacturer
Icon resin infiltration	Methacrylate-based resin (approximately 99%)	DMG Dental-Material Gesellschaft mbH, Hamburg, Germany
Icon Etch	15% hydrochloric acid	DMG Dental-Material Gesellschaft mbH, Hamburg, Germany
Icon Dry	99% ethanol	DMG Dental-Material Gesellschaft mbH, Hamburg, Germany
ZnO nanoparticles	ZnO nanopowder 99.8% (10-30 nm)	SkySpring Nanomaterials, Inc., Houston, Texas, United States
MgO nanoparticles	MgO nanopowder 99.9% (10-3 0 nm)	SkySpring Nanomaterials, Inc., Houston, Texas, United States
Methylene Blue	C_16_H_18_CIN_3_S.xH_2_O	Biochem Chemopharma Company, Cosne-Cours-sur-Loire, France

Thermocycling process

The specimens were incubated at 37°C and 100% humidity for 24 hours. Subsequently, all samples underwent thermocycling for 5,000 cycles. This process involved alternating immersion in baths at 5°C and 55°C, with each cycle consisting of a 30-second dwell time in each bath and a five-second transfer time [31,32].

Microleakage tests and assessment

The specimens were submerged in a 2% methylene blue solution at 37°C for 24 hours. Post-immersion, all specimens were rinsed with running tap water and sectioned buccolingually using a slow-speed handpiece disc. The sectioned samples were then analyzed under a stereomicroscope at 40x magnification (Optika Srl, Ponteranica, Italy). Microleakage was quantified by evaluating the extent of methylene blue penetration (MBP), according to the following criteria [23]: 0 = no MBP, 1 = MBP to outer half of enamel, 2 = MBP to inner half of enamel; 3 = MBP to outer half of dentin, and 4 = MBP to inner half of dentin.

Statistical analysis

Descriptive statistics, including mean and standard deviation, were calculated for each group to summarize the data. An analysis of variance (ANOVA) test was performed to determine if there were significant differences between the groups. Post-hoc comparisons were made using Tukey's Honest Significant Difference (HSD) test to identify specific group differences. The significance level was set at α=0.05.

## Results

XRD characterization

**Figure 2:**
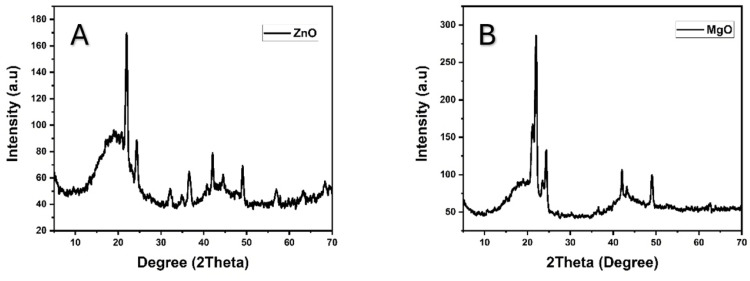
XRD patterns of ZnO (A) and MgO (B) nanoparticles. The peak characterizations confirm the crystalline nature and type of the nanoparticles. XRD: X-ray diffraction; ZnO: zinc oxide; MgO: magnesium oxide

The presence of distinct peaks confirms the crystalline structure of these powders, with no additional peaks observed, indicating the absence of impurities and confirming the high purity of the product. The XRD peaks correspond to the reference code ICSD 98-006-5122 for ZnO and ICSD 98-017-0905 for MgO nanoparticles (Figure 2).

MBP

**Figure 3:**
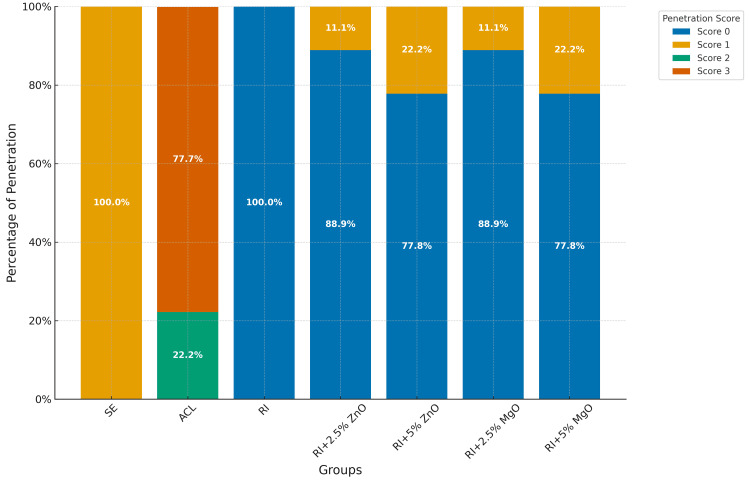
Percentages of methylene blue penetration of each group. ACL: artificially induced carious lesion; RI: resin infiltrate; ZnO: zinc oxide; MgO: magnesium oxide

RI group showed complete prevention of MBP, with a mean score of 0. ACL had the highest penetration scores, significantly differing from all other groups. Modifications with ZnO and MgO at 2.5% concentrations maintained high sealing efficacy with minimal penetration. Higher concentrations of ZnO and MgO NPs (5%) showed slightly higher penetration but were still effective (Table 2 and Figure 3).

**Table 2:** Significant relations between different groups for methylene blue penetration. ACL: artificially induced carious lesion; RI: resin infiltrate; ZnO: zinc oxide; MgO: magnesium oxide

Group 1	Group 2	Mean Difference	P-Value	Lower Bound	Upper Bound	Significant
ACL	RI	-2.7778	<0.001	-3.0839	-2.4716	*
ACL	RI+2.5% ZnO	-2.6667	<0.001	-2.9728	-2.3605	*
ACL	RI+2.5% MgO	-2.6667	<0.001	-2.9728	-2.3605	*
ACL	RI+5% ZnO	-2.5556	<0.001	-2.8617	-2.2494	*
ACL	RI+5% MgO	-2.5556	<0.001	-2.8617	-2.2494	*
RI	SE	-1.0000	<0.001	-1.3061	-0.6939	*
RI+2.5% ZnO	SE	-0.8889	<0.001	-1.1949	-0.5827	*
RI+2.5% MgO	SE	-0.8889	<0.001	-1.1949	-0.5827	*
RI+5% ZnO	SE	-0.7778	<0.001	-1.0839	-0.4716	*
RI+5% MgO	SE	-0.7778	<0.001	-1.0839	-0.4716	*
RI	RI+2.5% ZnO	-0.1111	0.9861	-0.4172	0.1949	-
RI	RI+2.5% MgO	-0.1111	0.9861	-0.4172	0.1949	-
RI	RI+5% ZnO	-0.2222	0.8332	-0.5283	0.0839	-
RI	RI+5% MgO	-0.2222	0.8332	-0.5283	0.0839	-
RI+2.5% ZnO	RI+5% ZnO	-0.1111	0.9861	-0.4172	0.1949	-
RI+2.5% MgO	RI+5% ZnO	-0.1111	0.9861	-0.4172	0.1949	-
RI+2.5% ZnO	RI+5% MgO	-0.1111	0.9861	-0.4172	0.1949	-
RI+2.5% MgO	RI+5% MgO	-0.1111	0.9861	-0.4172	0.1949	-

**Figure 4:**
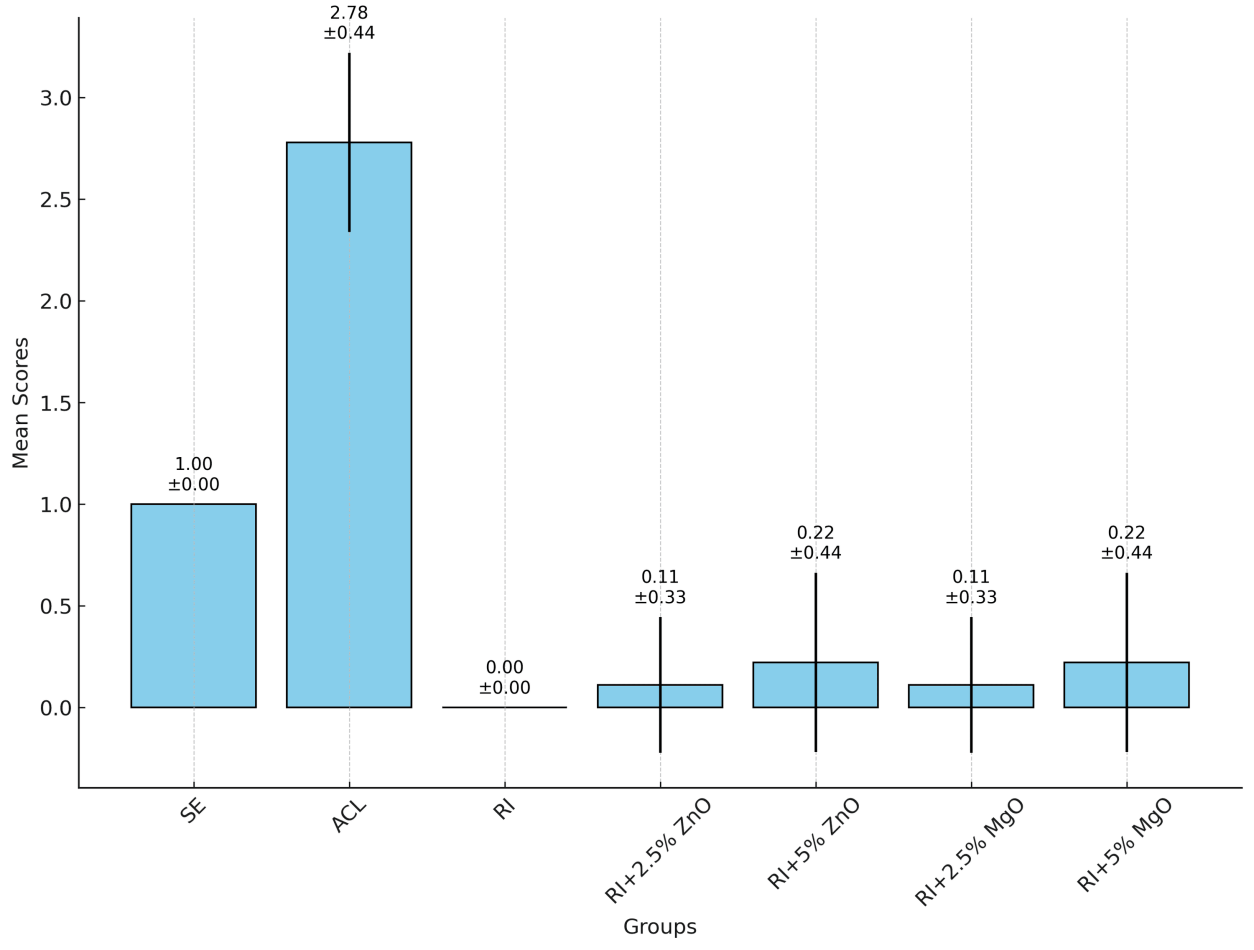
Mean MBP and standard deviation for each group. MBP: methylene blue penetration; ACL: artificially induced carious lesion; RI: resin infiltrate; ZnO: zinc oxide; MgO: magnesium oxide

A one-way ANOVA test was conducted to compare the mean scores among these groups (Figure 4). The results of the ANOVA test indicated that there were statistically significant differences between the groups (F = 230.09, p < 0.001). This indicates that the treatment type significantly affects MBP. Post-hoc analysis using Tukey's HSD test showed significant differences between several group pairs. Notably, the ACL group differed significantly from all other groups (p < .001). The RI group did not show significant differences when compared with RI+2.5% ZnO and RI+2.5% MgO groups, indicating similar effectiveness. However, significant differences were observed between the SE group and all treatment groups, including RI and its modifications.

## Discussion

In this study, the efficacy of RI modified with 2.5% and 5% ZnO and MgO nanoparticles in reducing microleakage on artificially induced enamel carious lesions was investigated. These nanoparticle modifications effectively decreased MBP, with significant differences observed compared to untreated lesions. This suggests that the incorporation of ZnO and MgO nanoparticles into RI enhances their sealing ability, particularly in enamel and dentin. The minimal MBP in treated samples emphasizes the potential of these modifications to improve the clinical performance of RI, potentially extending their longevity and reducing the risk of secondary caries formation.

The RI group demonstrated complete prevention of methylene blue leak, as evidenced by a mean penetration score of 0 across all samples. This indicates that the RI treatment effectively seals the ACL, preventing any microleakage. The superiority of RI is further corroborated by the significant differences observed between the RI group and the ACL group, as well as the SE group. Our findings align with those of Klaisiri et al., who reported 100% of MBP Score 1 on SE, attributed to the surface porosity of SE [23]. In the RI group, their study demonstrated 90% penetration into the outer enamel, a result that closely mirrors our findings, where MBP penetration was entirely inhibited. Another study has also shown an increase in the percentages of MBP of RI from 10% of outer enamel to 30% and 70% after 5000 and 10000 of thermocycling, respectively, after 24 hours of RI application [33]. This indicates that as the materials age, they are unable to maintain the sealing capacity of RI against oral fluids and bacterial invasion. In the current study, we used bovine enamel from incisor teeth, while other studies used human premolars to create ACLs. The difference in our results may be attributed to the fact that bovine teeth demineralize more quickly due to their softer and more porous nature compared to human enamel [34,35].

Both modifications with ZnO and MgO at 2.5% and 5% concentrations showed considerable effectiveness in preventing MBP. The RI+2.5% ZnO and RI+2.5% MgO groups exhibited mean penetration scores close to 0, with 88.89% of samples showing no penetration (score 0) and only 11.11% showing minimal penetration (score 1). These results highlight that the addition of ZnO and MgO at 2.5% concentrations did not compromise the sealing ability of the RI. However, the RI+5% ZnO and RI+5% MgO groups, while still effective, showed a slightly higher incidence of minimal penetration. With 77.78% of samples scoring 0 and 22.22% scoring 1, these findings suggest that higher concentrations of ZnO and MgO might marginally reduce the RI's sealing efficacy. This might be related to the increase in the viscosity of the RI as the concentration of the nanoparticles increases and the decrease in the degree of conversion, increase in the porosity of the RI, and hydrophilic properties of the nanoparticles incorporated into the RI [19-21,36]. The absence of significant differences between the 2.5% and 5% concentration groups indicates that both concentrations provide similar levels of sealing capabilities against microleakage, but the lower concentration might be slightly more optimal. Although RI has shown great sealing capacity, they might be frequently associated with secondary caries lesions as resin-based materials are susceptible to bacterial colonization [20,21]. The necessity of incorporating these nanoparticles is important to eliminate these drawbacks. In this regard, 2.5% of ZnO and MgO nanoparticles seem to be appropriate solutions between microleakage and anticaries properties.

The SE group consistently exhibited a mean score of 1, reflecting the natural resistance of intact enamel to MBP. In contrast, the ACL group had the highest mean score of 2.78, with significant MBP. This stark contrast highlights the vulnerability of untreated caries lesions and the critical importance of effective treatment. It is important to recognize that deep acid penetration into the tooth structure may require modifying RI to include antibacterial properties, in order to halt or prevent the progression of dental caries while maintaining its penetration capacity.

The significant differences observed in Tukey's HSD test between the ACL group and all treatment groups confirm the efficacy of RI and its modifications in preventing MBP. These differences also underscore the necessity of treating caries lesions to prevent further dental complications. The increase in microleakage properties with material aging [23] as stated by studies may lead to the development of secondary caries lesions; therefore, the incorporation of antibacterial nanoparticles may further halt or prevent secondary caries lesion developments.

Numerous thermocycling protocols ranging from 500 to 10,000 cycles have been utilized to test microleakage. However, 5000 cycles are often considered sufficient for simulating the aging of adhesive restorative materials [31,32,37]. In this study, we employed 5000 thermocycles, approximating about six months of clinical aging [31].

The findings of this study have important implications for clinical practice, particularly in the context of preventive dentistry. The RI, especially in its unmodified form, demonstrates exceptional sealing ability, making it a valuable tool for managing early caries lesions and increasing sealing efficiency between the margin of the restoration and cavity preparation margins [32]. The modified RI with ZnO and MgO also showed promising results especially 2.5%, suggesting potential for further optimization.

Future research should explore the long-term effects of these treatments and their performance under varying clinical conditions. Additionally, investigating other potential modifications or combinations could further enhance the antibacterial properties of RI. Understanding the underlying mechanisms by which ZnO and MgO nanoparticles influence the sealing properties of RI could also provide valuable insights for developing more effective dental materials.

Several limitations should be acknowledged in this study. Firstly, the research was conducted using bovine teeth with artificially induced carious lesions, which may not perfectly replicate the clinical conditions found in human teeth with natural caries. Additionally, the study focused solely on microleakage using MBP as an assessment method, whereas other factors influencing RI performance, such as bond strength and wear resistance, were not evaluated. Furthermore, the study only investigated two concentrations (2.5% and 5%) of ZnO and MgO nanoparticles in RI, limiting the exploration of potential dose-dependent effects or optimal NP formulations. Future research could address these limitations by conducting studies on human teeth, incorporating comprehensive mechanical testing, and exploring a wider range of NP concentrations and types to further elucidate their effects on RI properties.

## Conclusions

This study underscores the exceptional efficacy of RI in preventing MBP in ACL. The integration of ZnO and MgO nanoparticles at 2.5% and 5% concentrations preserves the RI's performance in sealing enamel and dentine. These results highlight the potential of MgO and ZnO-enhanced RI to enhance clinical outcomes by potentially extending restoration durability and reducing the risk of secondary caries. Further research is needed to validate these findings clinically and explore broader applications of nanoparticle-modified RI in dental practice.

## References

[1] Gomez J (2015). Detection and diagnosis of the early caries lesion. BMC Oral Health.

[2] Kassebaum NJ, Bernabé E, Dahiya M, Bhandari B, Murray CJ, Marcenes W (2015). Global burden of untreated caries: a systematic review and metaregression. J Dent Res.

[3] Featherstone JD (2008). Dental caries: a dynamic disease process. Aust Dent J.

[4] Machiulskiene V, Campus G, Carvalho JC (2020). Terminology of dental caries and dental caries management: consensus report of a workshop organized by ORCA and Cariology Research Group of IADR. Caries Res.

[5] Pitts NB, Zero DT, Marsh PD (2017). Dental caries. Nat Rev Dis Primers.

[6] Daruich PM, Brizuela M (2023). Remineralization of initial carious lesions. StatPearls [Internet].

[7] Todorova V, Filipov I, Petrova R (2020). In vitro comparison of several methods for initial proximal caries detection. Folia Med (Plovdiv).

[8] Gimenez T, Tedesco TK, Janoian F (2021). What is the most accurate method for detecting caries lesions? A systematic review. Community Dent Oral Epidemiol.

[9] Chan EK, Wah YY, Lam WY, Chu CH, Yu OY (2023). Use of digital diagnostic aids for initial caries detection: a review. Dent J (Basel).

[10] Al Saffan AD (2023). Current approaches to diagnosis of early proximal carious lesion: a literature review. Cureus.

[11] Lim ZE, Duncan HF, Moorthy A, McReynolds D (2023). Minimally invasive selective caries removal: a clinical guide. Br Dent J.

[12] Ericson D (2007). The concept of minimally invasive dentistry. Dent Update.

[13] Mazzitelli C, Josic U, Maravic T (2022). An insight into enamel resin infiltrants with experimental compositions. Polymers (Basel).

[14] Dziaruddin N, Zakaria AS (2022). Resin infiltration of non-cavitated enamel lesions in paediatric dentistry: a narrative review. Children (Basel).

[15] Meyer-Lueckel H, Paris S (2010). Infiltration of natural caries lesions with experimental resins differing in penetration coefficients and ethanol addition. Caries Res.

[16] Paris S, Dörfer CE, Meyer-Lueckel H (2010). Surface conditioning of natural enamel caries lesions in deciduous teeth in preparation for resin infiltration. J Dent.

[17] Tassery H, Levallois B, Terrer E (2013). Use of new minimum intervention dentistry technologies in caries management. Aust Dent J.

[18] Yu J, Huang X, Zhou X (2020). Anti-caries effect of resin infiltrant modified by quaternary ammonium monomers. J Dent.

[19] Peutzfeldt A, Asmussen E (2004). Determinants of in vitro gap formation of resin composites. J Dent.

[20] Beyth N, Domb AJ, Weiss EI (2007). An in vitro quantitative antibacterial analysis of amalgam and composite resins. J Dent.

[21] Zalkind MM, Keisar O, Ever-Hadani P, Grinberg R, Sela MN (1998). Accumulation of Streptococcus mutans on light-cured composites and amalgam: an in vitro study. J Esthet Dent.

[22] Zhou Y, Huang X, Wu L, Liang Y, Huang Y, Huang S (2023). Microleakage, microgap, and shear bond strength of an infiltrant for pit and fissure sealing. Heliyon.

[23] Klaisiri A, Janchum S, Wongsomtakoon K, Sirimanathon P, Krajangta N (2020). Microleakage of resin infiltration in artificial white-spot lesions. J Oral Sci.

[24] He Y, Ingudam S, Reed S, Gehring A, Strobaugh TP Jr, Irwin P (2016). Study on the mechanism of antibacterial action of magnesium oxide nanoparticles against foodborne pathogens. J Nanobiotechnology.

[25] Bhattacharya P, Dey A, Neogi S (2021). An insight into the mechanism of antibacterial activity by magnesium oxide nanoparticles. J Mater Chem B.

[26] Pushpalatha C, Suresh J, Gayathri VS (2022). Zinc oxide nanoparticles: a review on its applications in dentistry. Front Bioeng Biotechnol.

[27] Krishnamoorthy R, Athinarayanan J, Periyasamy VS (2022). Antibacterial mechanisms of zinc oxide nanoparticle against bacterial food pathogens resistant to beta-lactam antibiotics. Molecules.

[28] Kidd EA (1976). Microleakage: a review. J Dent.

[29] Hamalaw SJ, Kareem FA, Noori AJ (2023). Dispersion and demineralization inhibition capacity of novel magnesium oxide nanoparticles varnish on enamel surfaces against Streptococcus mutans (an in vitro study). Coatings.

[30] Freitas MC, Nunes LV, Comar LP, Rios D, Magalhães AC, Honório HM, Wang L (2018). In vitro effect of a resin infiltrant on different artificial caries-like enamel lesions. Arch Oral Biol.

[31] Carreira M, Antunes PV, Ramalho A (2017). Thermocycling effect on mechanical and tribological characterization of two indirect dental restorative materials. J Braz Soc Mech Sci.

[32] Tulunoglu O, Tulunoglu IF, Antonson SA, Campillo-Funollet M, Antonson D, Munoz-Viveros C (2014). Effectiveness of an infiltrant on sealing of composite restoration margins with/without artificial caries. J Contemp Dent Pract.

[33] Klaisiri A, Vongsang J, Leelaudom T, Krajangta N (2023). Methylene blue penetration of resin infiltration and resin sealant in artificial white-spot lesions. Eur J Dent.

[34] Amaechi BT, Higham SM, Edgar WM (1998). Factors affecting the development of carious lesions in bovine teeth in vitro. Arch Oral Biol.

[35] Lippert F, Lynch RJ (2014). Comparison of Knoop and Vickers surface microhardness and transverse microradiography for the study of early caries lesion formation in human and bovine enamel. Arch Oral Biol.

[36] Gnanasambandan P, Adjeroud N, Leturcq R (2022). Role of ZnO and MgO interfaces on the growth and optoelectronic properties of atomic layer deposited Zn1− xMgxO films. J Vacuum Sci Tech A.

[37] Pazinatto FB, Campos BB, Costa LC, Atta MT (2003). Effect of the number of thermocycles on microleakage of resin composite restorations. Pesqui Odontol Bras.

